# Comparison of Gait in Women with Degenerative Changes of the Hip Joint and Healthy Women Using the MoKA System—A Pilot Study

**DOI:** 10.3390/s24196417

**Published:** 2024-10-03

**Authors:** Maciej Kuś, Dagmara Wasiuk-Zowada, Magdalena Henke, Justyna Szefler-Derela, Andrzej Knapik

**Affiliations:** 1 Doctoral School, Faculty of Health Sciences in Katowice, Medical University of Silesia, 40-752 Katowice, Poland; maciejkus97@gmail.com; 2 Department of Physiotherapy, Faculty of Health Sciences in Katowice, Medical University of Silesia, 40-752 Katowice, Poland; jszefler@sum.edu.pl; 3 Student Scientific Club of Adapted Physical Activity and Sport, Faculty of Health Sciences in Katowice, Medical University of Silesia, 40-752 Katowice, Poland; magdahenke95@gmail.com; 4 Department of Adapted Physical Activity and Sport, Faculty of Health Sciences in Katowice, Medical University of Silesia, 40-752 Katowice, Poland; aknapik@sum.edu.pl

**Keywords:** hip osteoarthritis, 6 min walking test (6MWT), gait analysis, pelvic movements, movement kinematic analysis (MoKA)

## Abstract

Osteoarthritis (OA) is a global problem. There are few reports in the literature regarding the temporal and spatial parameters of gait in people with OA. The aim of this study was to determine spatiotemporal parameters for the pelvis and lower limbs during walking in women with OA and to compare these parameters with healthy people. For this purpose, a 6 min walking test (6MWT) was carried out. OA subjects had worse outcomes compared to the control group (*p* < 0.05). Data were collected using IMU sensors integrated into the MoKA system and mounted on indicator points on the body. Limited mobility of the pelvis in the frontal plane was observed in the study group, which influenced walking strategy. For the comparison with the control group at each minute, *p* < 0.05. IMU sensors attached to the body and integrated in one application provide extensive research and diagnostic capabilities.

## 1. Introduction

The most common form of joint disease and one of the main causes of pain in older people is osteoarthritis—OA [1]. OA is caused by complex biological and then physiological processes that lead to pain and limited functional capabilities of the patient [2,3]. The problem of OA is of global importance, with an estimated 240 million people struggling with this disease [1]. In developed societies, this problem may affect up to 20% of adult population [4,5]. Factors predisposing to the severity of symptoms of this disease include older age, female gender, excessive body weight, cartilage damage, abnormal joint biomechanics, and limited physical activity [6,7]. Pain and gait disturbances are the main symptoms of OA affecting the hip joints and knees, constituting the basis for clinical diagnosis and therapy [8,9]. In clinical practice, gait disturbances are most often assessed subjectively. The reason for this is the lack of expensive, complicated, specialized gait assessment equipment.

Previously, patients’ gait analysis tests were most often performed in laboratory conditions [10,11]. The costs of the examination, the number of people who can be examined, the short walking distance, and its forced pace limit the use of such analyzes in common diagnostics [12]. This prompted researchers to look for tools that would minimize these limitations, in the form of sensors worn on the body.

The first laboratory tests using accelerometers worn on the body were performed already in the 1970s [13,14]. Initially, the variability of movements was studied, and attempts were made to identify and classify them [15,16]. The next stage was combining sensors into systems, which created the possibility of greater precision in the description of movement [17,18]. Miniaturization and economization of energy consumption by sensors, thanks to technological progress, have created the possibility of using them in research and therapy in non-clinical conditions [14,19].

Research on the development of the possibilities of the qualitative and quantitative analysis of human movement is conducted in many ways. In addition to electromyography, which can be used in laboratory conditions [20,21], the use of a miniature gyroscope [22] or the use of coating traditional fabrics with piezoresistive, piezoelectric and piezocapacitive polymers has been proposed, which aims at provide information on gait parameters [23]. Technological advances provide increasing opportunities for the use of wearable sensors [24]. Among these sensors, inertial sensors are the most often used [25]. The argument for using this type of system is that gait testing under natural conditions may differ from artificial laboratory conditions. In addition, gait testing over a longer distance can reveal aspects such as a possible change in the spatiotemporal parameters of movement due to prolonged activity.

**Figure 1:**
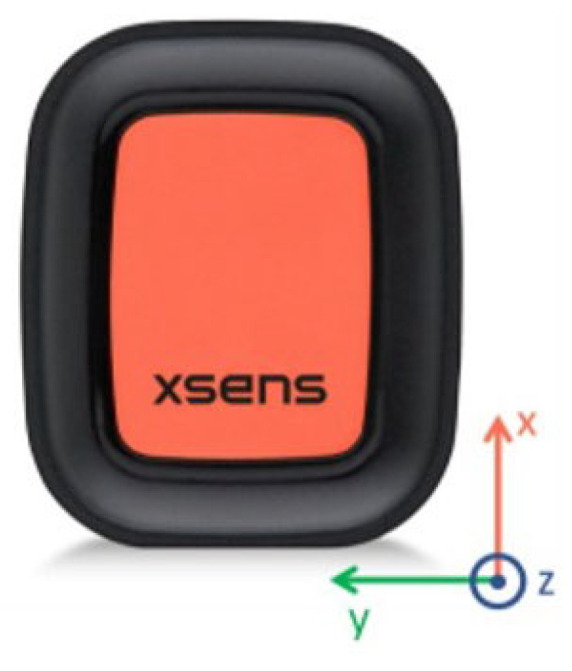
The sensor used during the study.

The idea of using inertial sensors in technology is several dozen years old. The combination of these sensors into one IMU (Inertial Measurement Unit) system, which was used in astronautics during the Apollo missions [26]. The miniaturization of the IMU made it possible to integrate a 3-axis accelerometer, gyroscope, and magnetometer, thus obtaining a lot of data regarding changes in the sensor’s position. This created opportunities to study human movements. IMUs can be combined into systems appropriate to their needs [27]. IMU sensors (Figure 1) carry out measurements of acceleration, angular velocity, and magnetic field strength at a high sampling rate in each of the three axes.

These measurements are processed by the sensor’s processor, using Kalman filters to form a quaternion that determines the sensor’s orientation in a three-dimensional space. The processed measurement results are sent via Bluetooth Low Energy (BLE) to a data concentrator. The data concentrator, in the course of the patient examination procedure, collects the transmitted measurement data, preprocesses them (converting quaternions into Euler angles), and sends them to the cloud storage at the end of the examination.

**Figure 2:**
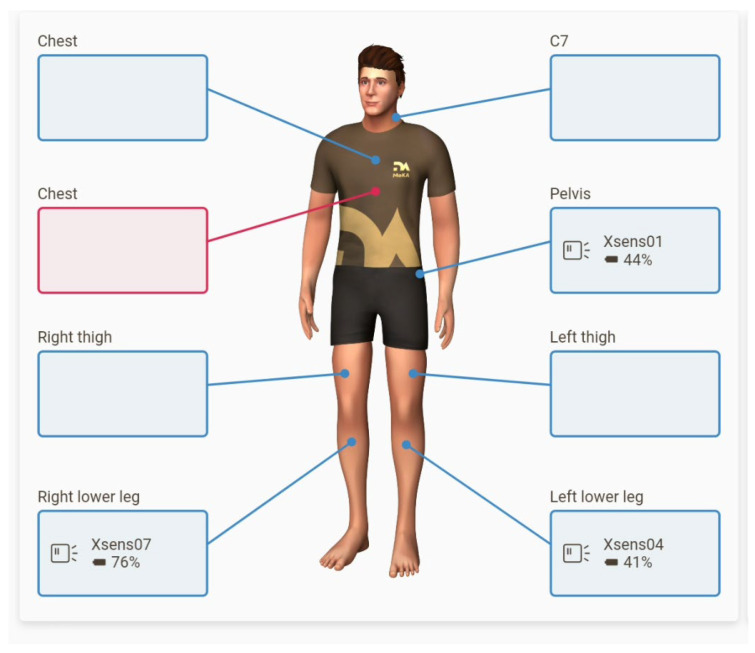
Positioning of sensors during the study.

The patient examination is carried out in two phases: calibration in habitual posture (Figure 2) and measurement during the patient’s gait. Based on the calibration data, the sensor orientation measurements are processed into a system linked to human body surfaces.

In further processing, time intervals for the constant gait direction are detected, and then the rotation statistics for each plane and each measured human body segment are determined.

Complementing them with a sensor measuring heart rate (HR) enables the collection of quantitative (exercise load) and qualitative (temporal and spatial parameters of movement) data [28]. MoKA, due to programmed algorithms, enables the calculation of data for research purposes, as well as in the future, for the needs of physiotherapy or recreation. The advantages of this system allow for the examination and assessment of many parameters in various patient groups. The global importance of OA and the small number of reports on the spatiotemporal parameters of gait in people with OA justify research in this direction.

The aim of the study was to determine the spatiotemporal parameters of the pelvis and lower limbs during gait in women with hip OA and to compare these parameters with the gait of healthy women.

## 2. Materials and Methods

### 2.1. Participants

Nineteen women took part in the study. All respondents were divided into two main groups: study group (SG) and control group (CG). There were 9 women in the SG with diagnosed degenerative changes in the hip joint, who were fully diagnosed and qualified for arthroplasty. In four women, this concerned the left hip (SGL), and in five women, the right hip (SGR). The control group (CG) consisted of 10 active women—volunteers, without any problems with the musculoskeletal system. The CG was recruited among people who regularly exercised in physical activities at the University of the Third Age.

### 2.2. Methods

Age data were collected, height and weight were measured, and BMI was calculated based on these data. All subjects performed the 6 min walking test (6MWT) in accordance with the adopted procedure [29]. Before the test, the subjects rested in a sitting position for 10 min and did not engage in intense exercise 2 h before the test. The test was performed in a long corridor, enabling participants to walk on a straight section of 32 m. The subjects were dressed in comfortable clothes and sports shoes.

During the test, the subjects had IMU sensors, integrated in the MoKA system, placed on their bodies. One of these sensors was placed directly on the sacrum using double-sided tape intended for contact with the skin. It enabled the ongoing measurement of pelvic movements in the sagittal, frontal, and transverse planes in angular values. In addition, the subjects had IMU sensors placed on their lower legs, directly under the tibial tuberosities. The task of these sensors was to measure limb movements in the frontal and transverse planes. The value of 0 was the resting position in the habitual position—determined individually for each subject. All deflections to the left were recorded as negative values, and all deflections to the right were recorded as positive values.

The calculations took into account measurements from one length of the corridor from the first, third and sixth minutes of walking. Recurrences were excluded to avoid measurement disturbances.

### 2.3. Ethics Approval

The study was approved by the Bioethics Committee of the Medical University of Silesia in Katowice (Decision no. BNW/NWN/0052/KB1/41/24; 21 May 2024) and conformed to the Helsinki Declaration. Written consent was obtained from all the individual participants included in the study.

### 2.4. Statistical Analysis

Descriptive statistics of ranges of motion (min–max) in individual planes were performed. Results from subsequent minutes were compared using repeated measures ANOVA. The correlations of age and morphological parameters with distance were calculated using Spearman’s correlation coefficients. Kruskal–Wallis ANOVA was used for intergroup comparisons.

## 3. Results

The analyzes did not reveal any differences between groups regarding basic morphological parameters. However, the 6MWT results differed significantly between the study groups and the control group. The influence of age and morphological parameters on the 6MWT result was moderate—Table 1.

The analysis of time–spatial parameters of the pelvis indicates the stability of measurements in subsequent minutes of walking in each group and in all planes—Table 2.

**Figure 3:**
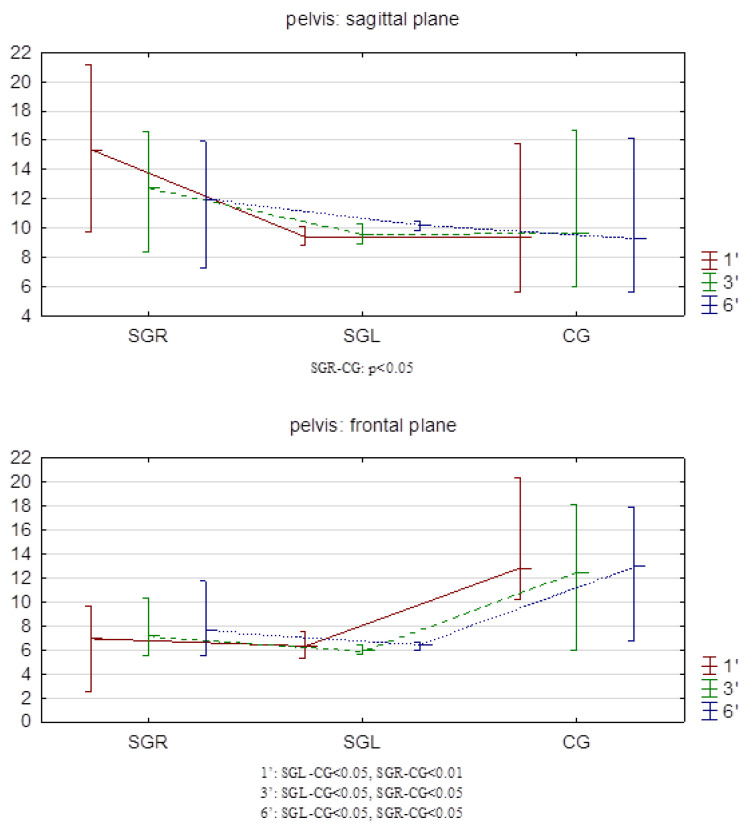
Ranges of pelvic motion in the sagittal and frontal planes.

Intergroup comparisons of the range of motion (max–min) of the pelvis in the sagittal plane showed differences in only one case. This concerned the first minute and the SGR–CGs. However, in the frontal plane, differences were noted in every minute of walking in both study groups and the control group (Figure 3).

Analysis of the spatiotemporal parameters of the lower limbs also indicates their relative stability over time. The percentage changes are greater than in the case of the pelvis, which is natural. In the transverse plane SGR, the high % at 6 min was probably a consequence of the small number of women tested. No statistically significant differences were found in intergroup comparisons—Table 3.

## 4. Discussion

Optical measurement of gait parameters, previously considered the gold standard of diagnostics, has a number of significant limitations [30]. The stationary nature of systems using cameras significantly limits the length of the gait section on which the measurement is performed and the duration of the test. It may also significantly affect the natural freedom of movement of the subjects [1,2,3]. The costs of the test, the possibilities regarding the number of people examined, and the need to be handled by properly trained specialists are other limitations.

The use of sensors worn on the body—IMU, integrated into one system, creates completely new possibilities. In MoKA (formerly known as SMART), it is possible not only to assess the spatiotemporal parameters of movement [31,32,33,34] at any walking distance, but also by measuring HR—exercise load [28]. Consequently, this makes it possible to assess the interrelationships between these variables.

Sensors worn on the body, integrated into one application, create opportunities for scientists to use these systems not only in laboratory conditions. Such systems can be widely used in diagnostics and therapy by doctors and physiotherapists. They can also be used for patients who, due to various restrictions, might have difficulty reaching research facilities. This applies, for example, to elderly people with frailty syndrome, children with celebratory palsy, or patients after amputations [35,36]. The practical use of these systems can also take place when selecting and assessing the quality of orthopedic supplies [37].

In addition to the benefits regarding both access to a larger number of different patient groups and the economics of research, in our opinion, it is important that the subjects can maintain natural and full freedom of movement, both in terms of their time and space parameters, as well as the dynamics and duration of activity. Another advantage of such applications is the ability to record objective data, which allow the patient’s current functional status to be monitored and subsequent therapeutic decisions to be made based on it. It should be noted that many countries are introducing IT systems in health care. These systems are not only e-prescriptions enabling the electronic flow of information on prescribed drugs between doctors, pharmacies, and insurers; many eHealth service platforms are patient-centric. They offer access to personalized information, providing electronic services for communication, self-care, and administrative tasks. This aims to realize the vision of patient-centered care, mobilizing citizens for self-care and prevention. This opens up prospects for improving the efficiency of health care provision and overcoming existing communication barriers [38,39].

The analysis of the presented research results indicates a relatively high stability of the tested parameters over time in all groups. On the one hand, this confirms the existence of individual gait stereotypes, and on the other hand, it indicates the accuracy of MoKA measurements. However, given the number of people examined, which is a limitation of this study, these observations should be treated with some caution. Therefore, further research is planned.

The selection of the CG was determined by the purpose of the study. Hence, there are no SG–CG differences regarding age and morphological parameters. Significant differences in distance are justified by SG’s problems with OA and a lower level of activity. The small negative impact of age and BMI on the 6MWT results also seems obvious (Table 1).

Researchers do not agree on the impact of overweight and obesity on the development of degenerative changes. On the one hand, the available literature clearly states that body weight and BMI do not directly influence the development of degenerative joint changes [4]. On the other hand, obesity has been correlated as a risk factor for years, mainly due to increased joint loads [40,41,42]. The repeated pathological loading of joints may, however, increase the value of compressive forces inside the joint, which may generate micro-damages to the hyaline cartilage [5]. Another risk factor for OA is obesity-related metabolic disorders [43]. The development of degenerative changes and metabolic diseases has a similar course, causing chronic low-intensity inflammation [44,45]. Both increased levels of cytokines and disturbed lipid metabolism are increasingly correlated with the development of osteoarthritis [46,47]. Moreover, hyperlipidemia and increased cholesterol levels may affect the remodeling of tissues around the articular cartilage, leading to the development of degenerative changes [48]. Following this, it can be concluded that increased body weight may indirectly contribute to the occurrence of degenerative changes in joints. However, the amount of material examined here does not confirm this thesis.

The key observation from this study is decreased pelvic mobility in the frontal plane in SG. Smaller maximum values in this plane are also noticeable—compared to CG. The relationship between degenerative changes and limited functional capabilities is evident and has been previously noted [6]. Limited mobility in this plane is a manifestation of adaptive changes—the adoption of a modified walking strategy by patients with OA. These people walk on a wider base combined with a shorter stride with a faster cadence. This is to minimize pain and reduce the possible risk of falls. The presented results are consistent with those of Ibar et al. [49]. These authors conducted similar studies on a group of women with OA of the hip (n = 11), comparing this with a control group (n = 11). In the cited study, IMUs were not placed on the sacrum, as in our study, but on the lateral side of the thigh between the femoral condyle and greater trochanter (thigh), on the dorsal side between both posterior superior iliac spines (pelvis), and on the dorsal side at the first lumbar spinous process (lumbar). There, the greatest differences between the groups were also observed in the lateral movements of the pelvis (frontal plane). This phenomenon can be explained by the reduced strength of the hip abductor muscles, whose function is also to stabilize the pelvis. Hence, people with OA limit the lateral movement of the pelvis to compensate for the weakness of the hip abductors.

In the present study, the mobility of the pelvis in the sagittal plane differed only in one case. This concerned the SGR-CG comparison and concerned only the measurement from 1 min of walking. This may indicate some similarity with the observations of Boekesteijn et al. These authors showed that in patients with OA of the hip joints, the mobility of the lumbar spine in the sagittal plane is greater than in healthy people [50]. This may indicate that patients choose compensatory gait strategies, which involve equalizing the position of the center of gravity on the support plane by changing the position of higher body sections [51]. The verification of this thesis, however, requires tests that also include the measurements of mobility of the trunk and cervical section.

No differences were found in intergroup comparisons of the lower limbs. This seems to justify the hypothesis that the ilio–lumbar–pelvic complex is crucial for body posture, both in static and dynamic conditions.

### Limitations

To the authors’ knowledge, this is the first study on gait analysis using IMU sensors in people with OA. The limitations of this study are typical of this type of research. Group sizes were limited due to the pilot nature of the study and the specificity of the study group—people immediately prior to hip replacement surgery (women). Further research is underway to verify the initial observations. However, this may constitute an impulse for further research, taking into account variables such as pain and the range of active and passive mobility of the examined joints.

## 5. Conclusions

Women with hip OA experience limitations in pelvic mobility in the frontal plane. The consequence is a different gait strategy than in healthy people. The use of sensors worn on the body (IMU), integrated into systems appropriate to their needs, creates wide research, diagnostic, and therapeutic possibilities.

## Figures and Tables

**Table 1:** Descriptive statistics of age, morphological parameters, and 6MWT.

Parameter	Group	Mean	Min–Max	*p*	R-Distance
Age [years]	SGL	71.33	69–73		−0.35
	SGR	65.83	60–78		−0.30
	CG	67.60	60–79		−0.35
Height [cm]	SGL	71.33	164–166		
	SGR	163	152–174		
	CG	160	148–177		
Weight [kg]	SGL	86	84.0–88.0		
	SGR	81.50	67.0–96.0		
	CG	69.26	57.4–88.9		
BMI	SGL	31.98	30.2–32.2		−0.31
	SGR	30.79	24.6–36.8		
	CG	27.46	22.1–35.6		−0.39
6MWT distance [m]	SGL	284.50	227–374	SGR-CG: <0.05 SGL-CG: <0.01	
	SGR	269.10	210–367		
	CG	469.50	400–576		

Abbreviations: SGL—study group left hip; SGR—study group right hip; CG—control group; 6MWT—6 min walking test.

**Table 2:** Spatial and temporal parameters of the pelvis during walking.

Group	Time	Pelvis								
		Sagittal Plane			Frontal Plane			Transverse Plane		
		Min–Max	Change: Max–Min		Min–Max	Change: Max–Min		Min–Max	Change: Max–Min	
			°	%		°	%		°	%
I. SGR	1′	20.1–29.5	9.4		−3.8–2.6	6.3		−11.0–10.0	21.0	
	3′	20.0–29.6	9.6	2.1	−3.7–2.3	6.0	4.7	−12.9–11.1	24.0	1.5
	6′	19.9–30.1	10.2	8.5	−4.1–2.4	6.4	1.6	−13.0–13.1	26.1	2.5
	average		9.7	5.5		6.2	3.2		23.7	2.0
II. SGL	1′	22.5–37.9	15.4		−3.9–3.0	6.9		−11.5–11.9	23.4	
	3′	21.5–34.3	12.8	1.7	−3.9–3.3	7.2	4.3	−14.6–14.1	28.7	22.6
	6′	21.8–33.8	12.0	2.2	−4.6–3.0	7.6	10.1	−9.9–12.7	22.6	3.4
	average		13.4	2.0		7.2	7.2		24.9	9.6
III. CG	1′	19.1–28.5	9.3		−6.4–6.5	12.8		−11.3–11.4	22.7	
	3′	19.2–28.8	9.6	3.2	−3.3–9.1	12.5	2.3	−10.8–12.5	23.3	2.6
	6′	19.2–28.5	9.3	0	−3.7–9.3	13.0	1.6	−12.5–11.5	24.0	5.7
	average		9.4	1.6		12.8	2.0		23.3	4.2

**Table 3:** Spatial and temporal parameters of the lower limbs during walking.

Group	Time	Left Lower Limb					
		Frontal Plane			Transverse Plane		
		Min–Max	Change: Max–Min		Min–Max	Change: Max–Min	
			^o^	%		^o^	%
I. SGR	1′	−6.9–8.3	15.2		−35.4–12.2	47.6	
	3′	−6.0–8.6	15.6	2.3	−19.9–25.8	45.7	3.9
	6′	−6.1–9.2	15.3	0.7	−6.3–8.8	15.1	68.2
	average		15.4	1.5		36.1	36.1
II. SGL	1′	−12.4–9.6	22.0		−14.4–13.4	27.8	
	3′	−13.3–10.1	23.4	6.4	−12.2–22.0	34.2	23.0
	6′	−13.8–10.4	24.4	10.9	−22.6–5.7	28.3	1.8
	average		23.3	8.7		30.1	12.4
III. CG	1′	−8.4–10.3	18.7		−15.9–16.3	32.2	
	3′	−8.5–12.0	20.5	9.6	−22.6–11.6	34.2	6.2
	6′	−9.0–12.0	21.0	12.3	−30.9–5.8	36.7	14.0
	average		20.1	11.0		34.4	10.1
		**Right lower limb**					
I. SGR	1′	−8.8–7.2	16.0		−13.7–16.8	30.5	
	3′	−10.9–6.3	17.2	7.5	−23.4–8.8	32.2	5.6
	6′	−10.5–6.2	16.7	4.4	−45.1–−12.0	33.1	8.5
	average		16.6	6.0		31.9	7.1
II. SGL	1′	−7.4–7.2	14.6		−12.5–17.9	30.4	
	3′	−7.1–7.3	14.4	1.4	−11.4–24.2	35.6	17.1
	6′	−6.9–6.5	13.4	8.2	−0.4–30.7	31.1	2.3
	average		14.1	4.8		32.4	9.7
III. CG	1′	−9.5–11.0	20.5		−12.8–18.5	31.3	
	3′	−9.1–11.2	20.4	0.5	−15.1–21.7	36.8	17.6
	6′	−8.9–12.7	21.6	5.4	−12.8–25.1	37.9	21.1
	average		20.8	3.0		35.3	19.4

## Data Availability

The raw data supporting the conclusions of this article will be made available by the authors on request.
